# Enlargements of the Spleen—V

**Published:** 1908-08-22

**Authors:** 


### ENLARGEMENTS OF THE SPLEEN—V.

### TUMOURS THAT MAY BE MISTAKEN FOR ENLARGED SPLEEN (*continued*).

*Gastric Growth.*—Malignant disease of the fundus of the stomach may be mistaken for enlargement of the spleen, especially in those very rare cases in which the stomach is affected by primary sarcoma. The latter tends to invade the whole of the organ, instead of forming a lump at one spot, as carcinoma usually does, and the result may be an enormous tumour occupying the left upper abdomen. The chief points which will help to distinguish the gastric tumour from an enlarged spleen are:—

(*a*) The mass may shift its position during the course of an examination, and from day to day.

(*b*) It does not present a sharp, well-defined edge.

(*c*) It may extend a considerable distance to the right of the middle line, although its lower limit may not be below the level of the umbilicus.

(*d*) It may be resonant on percussion in front.

(*e*) There may be well-marked gastric symptoms.

(*f*) There may be anæmia and some leucocytosis, but a differential leucocyte count would show a very small number of myelocytes, or none at all, which would exclude spleno-medullary leuchæmia, and an absence of extreme lymphocytosis would exclude lymphatic leuchæmia.

A man, aged 27, was admitted to hospital for an abdominal tumour and for loss of weight. He had lived in a very low-lying and badly drained part of Kent, where, when he was a child, ague was fairly common. When a school-boy he had suffered from attacks of fever, which appeared to have been ague. He had been married three years, and had three healthy children. Three months before his admission he began to feel out of sorts, and he noticed a pain in the upper part of the left side of the abdomen. A month later he noticed a lump in the situation of the pain. Five weeks before admission he took to his bed, and rapidly became worse. His usual weight was 10 st. 11 lb., but ten days before admission it was only 8 st. 13 lb. His bowels had been constipated, and he had suffered from sickness, but there had been neither melæna nor hæmatemesis. On admission he was very thin, anæmic, and in great pain, which was continuous, but with occasional colicky exacerbations. There was a firm tumour of not quite uniform consistence, situated to the left of the umbilicus, extending from the costal margin to within 2½ inches of the pubes and reaching backwards into the left loin, but not entirely filling it out. It moved downwards on inspiration. The outline of the tumour was not at all unlike that of a big spleen, except that there was no definite notch in its anterior border. A blood examination showed a leucocytosis, there being one white corpuscle to every hundred red. Shortly after admission an exploratory laparotomy was performed, and the tumour was found to be a malignant growth of the stomach. Previous to this the lump had been regarded as possibly splenic. The patient died, and at the post-mortem examination the stomach was found to be greatly enlarged, and infiltrated from end to end with lumpy soft white growth which on microscopical investigation proved to be a sarcoma. It was firmly adherent to the left lobe of the liver, to the spleen, diaphragm, splenic flexure of the colon, and pancreas. The stomach, pancreas, and spleen with adherent secondary growths weighed 9½ lb.

*The Differential Diagnosis of Cases of Enlarged Spleen.*—Having concluded that the tumour is an enlarged spleen, the next consideration will be to determine the nature of that enlargement; and in order to do this a knowledge of the possible causes of an increase in the size of that organ, and of the symptoms and physical signs which are associated with each, is necessary. A fairly complete list of the causes of splenic enlargement is given in the first article of this series, and it is not proposed to discuss every one separately; we shall altogether leave out typhoid, typhus, and relapsing fevers, erysipelas, septicæmia, puerperal fever, variola, scarlet fever, diphtheria, influenza, pneumonia, anthrax, and tuberculosis. The remainder we shall briefly discuss.

In the first place, a complete blood examination is essential to the proper making of a diagnosis in each case. It is necessary to insist, however, upon the fact that there are many conditions of enlarged spleen in which there is nothing distinctive about the blood, and the counts in such cases serve only in excluding those other conditions in which the blood changes are distinctive or pathognomonic. One is asked sometimes to examine the blood to see if a particular case is one of Hodgkin's disease; now the blood changes in Hodgkin's disease are entirely negative; the examination cannot possibly enable one to say "This *is* a case of Hodgkin's disease." The most it can do is to exclude such conditions of leuchæmia, the answer which the blood count enables one to give being: "The blood shows no decided changes, therefore this *may be* a case of Hodgkin's disease." If pathognomonic blood changes were present the condition could not be Hodgkin's disease; but if the blood changes are indefinite Hodgkin's disease remains in as one of the possibilities. Similarly in regard to certain other splenic enlargements.

(*To be continued.*)

